# Exploring subungual onycholemmal cysts: A rare case report and comprehensive literature review

**DOI:** 10.1002/ccr3.8734

**Published:** 2024-04-12

**Authors:** Toktam Safari Giv, Mahdiyeh Movahedi, Sahar Dadkhahfar, Farsad Biglari, Azadeh Rakhshan, Ghazal Mardani, Meisam Jafari Kafiabadi

**Affiliations:** ^1^ Skin Research Center Shahid Beheshti University of Medical Sciences Tehran Iran; ^2^ Shahid Beheshti University of Medical Sciences Tehran Iran; ^3^ Department of Orthopedic Surgery, Clinical Research Development Unit of Shohada‐e‐Tajrish Hospital Shahid Beheshti University of Medical Sciences Tehran Iran; ^4^ Department of Pathology, Shohada‐e‐Tajrish Educational Hospital, School of Medicine Shahid Beheshti University of Medical Sciences Tehran Iran

**Keywords:** nail, nail surgery, nail tumor, onycholemmal cyst

## Abstract

Subungual Onycholemmal Cyst (SOC) is a rare nail abnormality with different clinical presentations which can mimic different nail malignancies, such as melanoma, SCC, or glomus tumor. It is necessary for dermatologists and dermatopathologist to be aware of this pathology to make the proper diagnosis and treatment. SOC is a rare nail abnormality which affects the dermis of the nail bed. SOC has different clinical presentations, including onychodystrophy, ridging, clubbing, thickening, pigmentation, or even normal appearance. It can mimic different nail malignancies, such as melanoma, SCC, or glomus tumor. In this report, we describe a 54‐year‐old man with unilateral second right finger nail onychodystrophy and onycholysis for 1 year. He did not have any history of recent trauma, pain, or bleeding. It was completely resected by surgery. Nail biopsy can contribute to the early diagnosis of SOC and improvement of treatment outcomes.

## 1 INTRODUCTION

Subungual onycholemmal cysts (SOC) also known as subungual epidermoid inclusion cyst is an uncommon nail abnormality which affect the dermis of the nail bed. SOC has variable clinical presentations include onychodystrophy, ridging, clubbing, thickening, pigmentation, or even appears normal.^1^ It can also mimic different nail malignancy such as melanoma, SCC, or glomus tumor.^2^ In this report we document a case of unilateral second right finger nail onychodystrophy in 54‐year‐old man.

## 2 CASE HISTORY

A 54‐year‐old taxi driver was referred to our orthopedic department with onychodystrophy on a nail of the second right finger from a year before the current presentation. No history of recent trauma, pain, or bleeding has been noted. On physical examination, onycholysis and onychodystrophy of the right second nail were revealed. The lesion had tenderness when it was compressed bilaterally.

## 3 DIFFERENTIAL DIAGNOSIS, INVESTIGATIONS, AND TREATMENT

The differential diagnosis of SOC include subungual keratoacanthoma, squamous cell carcinoma (SCC), verrucous carcinoma (VC), glumus tumor, subungual metastasis, and onycholemmal carcinoma. It was resected by surgery.

**FIGURE 1:**
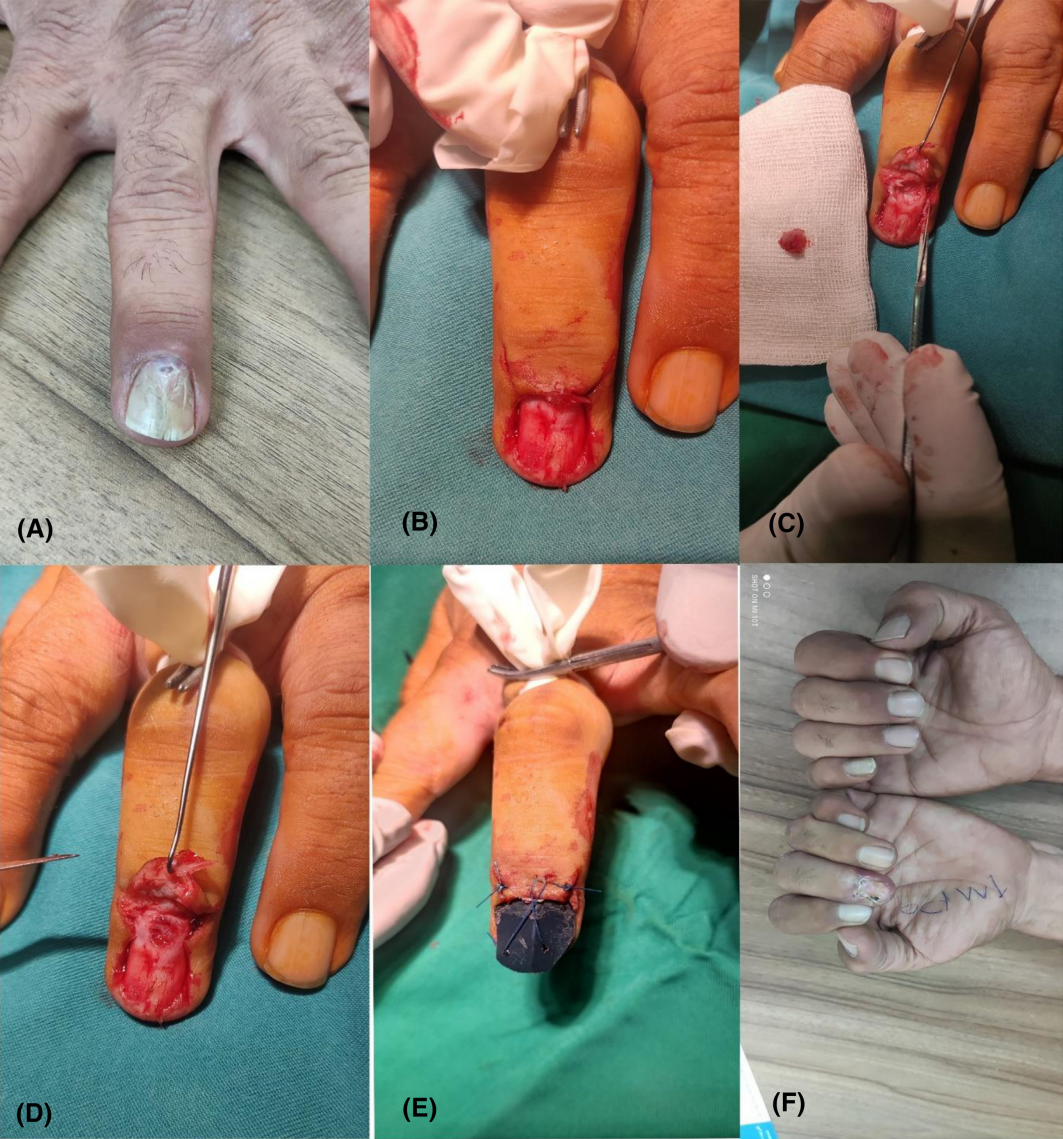
(A) Onycholysis and onychodystrophy of the right second nail; (B–E) surgical resection process; (F) One month after surgery.

**FIGURE 2:**
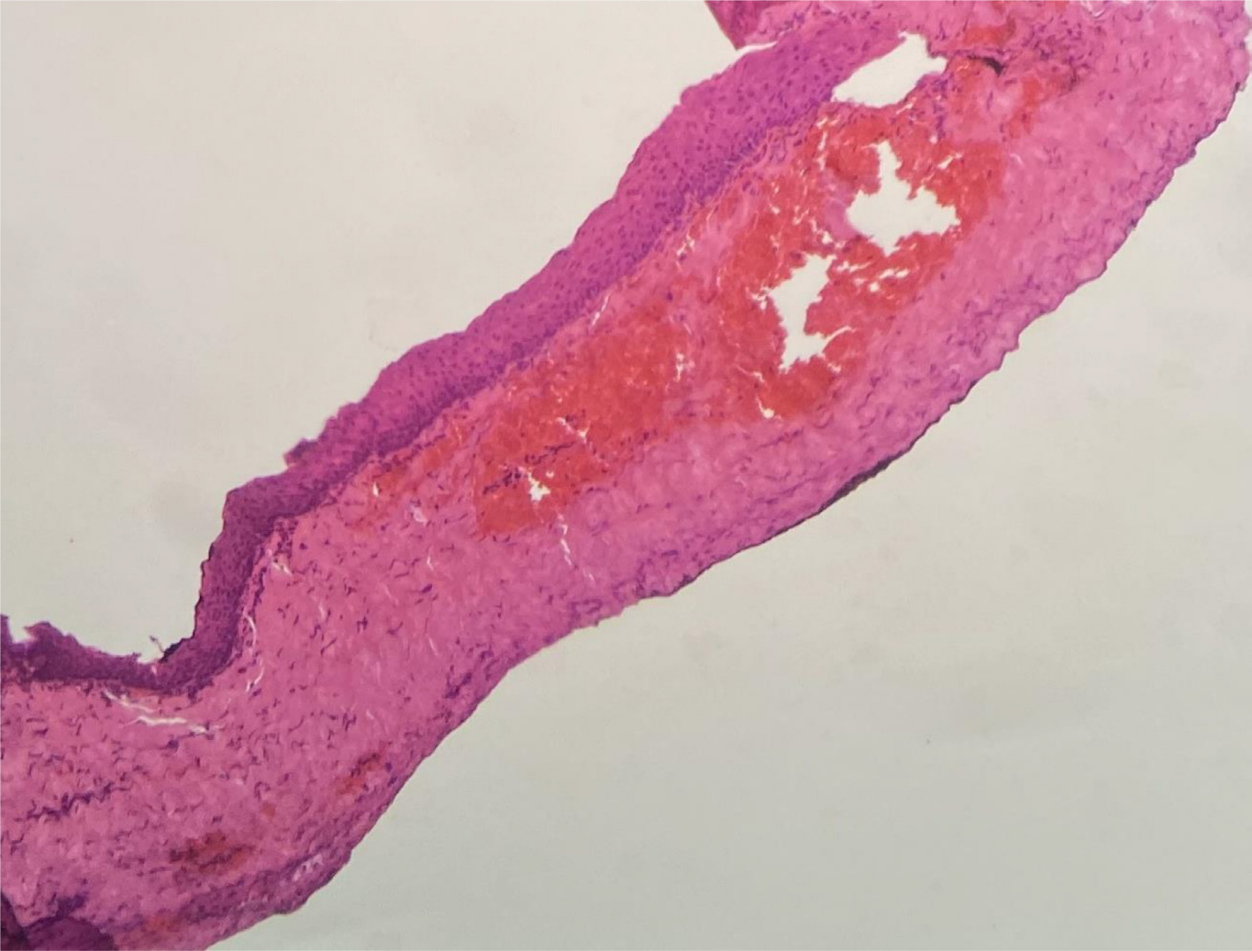
Onycholemmal cyst lining is composed of stratified squamous epithelium without granular layer supported by a thin fibrous connective tissue (×40, H&E staining).

Complete surgical excision of the nail was performed with local anesthesia (Figure 1). On surgery of the nail plate, a lesion measuring 10 × 10 mm appeared within the nail bed. The histopathological examination revealed a SOC (Figure 2).

## 4 OUTCOME AND FOLLOW‐UP

Following the complete surgical excision of the nail, the patient experienced resolution of symptoms associated with the SOC. Postoperative follow‐up examinations after 6 months and 1 year after revealed satisfying wound healing and restoration of nail integrity. The patient reported no recurrence of onychodystrophy or onycholysis, and no additional complications were noted.

Long‐term follow‐up care included routine clinical assessments to monitor for any signs of recurrence or development of new nail abnormalities. The patient was advised on proper nail care practices and encouraged to report any changes or concerns promptly.

## 5 DISCUSSION

SOCs are a rare, usually asymptomatic, nail abnormalities which are diagnosed incidentally in the progress of investigating other diseases like melanomas. Previous studies have described the possibility of an association between SOC and trauma, clubbing, and subsequently cyst formation. Clubbing can affect the dermis of the nail by fibroblast proliferation which causes cyst formation. Age, sex, lesion site, differential diagnosis (macroscopic and microscopic), underlying diseases, and treatments are listed in Table 1 of previous cases in this field.

**TABLE 1:** Age, sex, lesion site, differential diagnosis (macroscopic and microscopic), underlying diseases, and treatments of previous cases.

Patient no.	Age(years)/sex	Location	Clinical differential diagnosis	Histopathological differential diagnosis	Underlying condition	Treatment	First author reference number
Case 1	73/female	Left thumb nail	Infection	Invasive carcinoma with onycholemmal features.	Diabetes and hypertension History of working in farm/ field	Distal digital amputation	Rashid^7^
Case 2	45/female	Great toenail	Subungual melanoma	Subungual onycholemmal cyst	Nail trauma in childhood	Secondary intention with moist wound treatment	Lydrup^3^
Case 3	23/female	Right great toenail	Subungual malignant melanoma	Subungual onycholemmal cyst	5‐year pigmentation of nail with no clear underlying condition	Secondary intention	Busquets^2^
Case 4	70/male	Little finger of the right hand	SCC of the nail bed	Onycholemmal carcinoma	Not mentioned	Resection of tumor and diarticulation of distal phalanx	Inaoki^8^
Case 5	72/female	Left thumb nail	Bowen's disease SCC Proliferating onycholemmal tumor Wart/subungual exostosis	Onycholemmal horn	None	Surgical resection	Rodriguez^9^
Case 6	69/male	Nail bed of fifth right finger	Not mentioned	Malignant proliferating onycholemmal cyst	None	Amputation and disarticulation of involved phalanx	Alessi^6^
Case 7	77/male	Left second finger	SCC Metastatice carcinoma BCC	Primary onycholemmal carcinoma	Stage‐4 non‐small cell lung cancer/4‐year history of slow‐growing mass on left second finger	Involved nail amputation	Han^10^
Case 8	73/female	Right great toenail	Onychomycocis Trauma / Psoriasis/ Lichen planus	Calcified subungual epidermoid inclusion	7‐year thickening of nail bed	Nail plate avulsion and partial normalization of nail apparatus	Telang^1^

SOCs may have different clinical presentations, including onycholysis, onychodystrophy, pigmentation of the nail bed, ridging, and thickening. It most commonly affects single digits, mainly thumbs and great toenails, and pain is not uncommon.^3^

The subungual epidermoid inclusions that Lewin first referred to as the follicular microcysts of the nail bed are bulbous proliferations of the extremities of rete ridges, occasionally with the development of microcysts.^4^ These microcysts seldom lose their attachment to the nail bed epithelium and appear superficially within the dermis. The production of homogeneous keratin without a granular layer characterizes the keratinization of this superficial epithelial inclusion. The cyst's epithelium also mimics the follicular isthmus.^4^

Onycholemmal is the new term that is being used in literature. It depicts a specific type of subungual tumor with a pattern of onycholemmal microcysts and trichilemmal keratinization.^5^ All of these results point to the presence of vestiges of follicular units in the nail bed epithelium. In contrast to palms and soles, the nail epithelium is an invagination of the dorsal epidermis overlaying the digit that includes a few hair germs, consistent with embryology. The clinical presentation in our index case included onychodystrophy and onycholysis on a nail of the second right finger with no history of recent trauma, pain, or bleeding. Subungual melanomas and onycholemmal carcinomas can mimic SOC presentations and nail bed biopsy is required for appropriate diagnosis.^2^

On histopathology, onycholemmal cysts originate from the nail bed epithelium and are restored with eosinophilic keratin in the absence of a granular layer. The follicular isthmus outer root sheath is homologous to SOC and it is keratinized with no granular layer.^6^ The nail bed biopsy with partial or total nail avulsion is required for appropriate diagnosis.

The differential diagnosis of onycholemmal cyst include subungual keratoacanthoma. SCC, VC, glumus tumor, subungual metastasis, and onycholemmal carcinoma. The characteristic histological findings can help the exact diagnosis of these lesions.

There are no specific treatment recommendations.^3^ The biopsy from the affected area revealed multiple free‐lying cysts within the dermis of the nail bed, and in the near region to the epithelium of the nail bed. The cysts were lined by the stratified squamous epithelium with no granular layer or any cellular atypia. The cysts included luminal onycholemmal keratin.^3^

This report highlighted the variable clinical presentations of SOC, which can mimic different nail malignancies, including subungual melanomas, and onycholemmal carcinomas. Early diagnosis of SOC by nail biopsy can improve the treatment outcome.

## AUTHOR CONTRIBUTIONS

**Toktam Safari Giv:** Conceptualization; investigation; project administration; writing – review and editing. **Mahdiyeh Movahedi:** Conceptualization; data curation; writing – original draft; writing – review and editing. **Sahar Dadkhahfar:** Supervision; writing – review and editing. **Farsad Biglari:** Data curation. **Azadeh Rakhshan:** Visualization; writing – review and editing. **Ghazal Mardani:** Software. **Meisam Jafari Kafiabadi:** Conceptualization; data curation; writing – review and editing.

## FUNDING INFORMATION

No funding was received for this study.

## CONFLICT OF INTEREST STATEMENT

The authors declare that they have no conflicts of interest.

## CONSENT

Written informed consent was obtained from the patient for the publication of this case report and the accompanying images.

## Data Availability

The data are available from the author upon reasonable request.
